# Authors’ Reply: A Use Case for Generative AI in Medical Education

**DOI:** 10.2196/58370

**Published:** 2024-06-07

**Authors:** Tricia Pendergrast, Zachary Chalmers

**Affiliations:** 1 Department of Anesthesiology, University of Michigan Medicine, Ann Arbor, MI, United States; 2 Feinberg School of Medicine, Northwestern University, Chicago, IL, United States

**Keywords:** ChatGPT, undergraduate medical education, large language models

We thank the authors for their thoughtful comments on our paper titled, “Anki Tagger: A Generative AI Tool for Aligning Third-Party Resources to Preclinical Curriculum” [1,2]. The authors’ discussion of the ethical issues and limitations of generative artificial intelligence is both timely and important. As the capabilities of ChatGPT and other similar tools evolve, so must our conversations about the use of generative artificial intelligence in medicine and medical education.

With respect to the production of educational materials for medical trainees, ChatGPT’s ability to “hallucinate” and thereby provide misinformation should be of particular concern to educators. For example, when asked to summarize the research output of 50 scientists and cite relevant literature related to Chagas disease, ChatGPT made a major error in 86.7% of its outputs [3]. The problem of hallucination is more pronounced with smaller training data sets and may therefore disproportionately affect medical education content related to rare diseases, which are emphasized in licensing examinations. The problem of hallucination remains a substantial barrier to the widespread use of generative artificial intelligence in medical education.

We circumvented the issue of hallucination by embedding existing Anki flashcard decks in a large language model, rather than prompting ChatGPT to generate flashcards de novo from scientific literature [1]. Anki flashcard decks are among the third-party resources used by medical students to bridge perceived gaps in school curricula, especially regarding preparation for the USMLE (United States Medical Licensing Examination). Medical students report feeling overwhelmed with the number of third-party resources at their disposal and experience tension between these resources and their in-house curricula [4]. Their educators experience tension among different domains of responsibility including clinical practice, research, professional development, and education [5]. Therefore, it is beneficial to both teachers and students for medical education to be as efficient as possible. To this end, ChatGPT can organize and stratify third-party learning resources by relevance to lectures and other curricular elements [1].

While the integration of third-party resources into lesson plans for undergraduate medical education may be controversial, it is important to note that medical students are already using third-party resources instead of lectures by clinical educators [4]. Instead of viewing these learning materials as competition, our application of ChatGPT suggests the possibility of integrating third-party resources into existing medical curricula. Future studies should examine the impact of such an intervention on medical students’ academic performance and satisfaction as well as medical educator burnout.

## Abbreviations

USMLE: United States Medical Licensing Examination
